# Predicting peri‐implantitis incidence and implant failure via risk‐assessment and prognostication tools: A validation study

**DOI:** 10.1002/jper.70047

**Published:** 2025-12-26

**Authors:** Muhammad H. A. Saleh, Era Kakar, Giuseppe Troiano, Hamzeh Almashni, Jonathan Misch, Fariba Esperouz, Shahad Alhazmi, Hom‐Lay Wang, Zoltán Baráth, Istvan A. Urban

**Affiliations:** ^1^ Department of Periodontics and Oral Medicine University of Michigan School of Dentistry Ann Arbor Michigan USA; ^2^ Department of Medicine and Surgery University LUM Casamassima Italy; ^3^ Department of Clinical and Experimental Medicine University of Foggia Foggia Italy; ^4^ Department of Periodontology University of Szeged Szeged Hungary; ^5^ Graduate Implant Dentistry Loma Linda University Loma Linda California USA; ^6^ Urban Regeneration Institute Budapest Hungary

**Keywords:** dental implants, implant survival, peri‐implantitis, prognosis, retrospective studies, risk assessment

## Abstract

**Background:**

Identifying individuals at high risk for developing peri‐implantitis (PI) and then determining the prognosis for implants with PI is crucial for treatment planning.

**Methods:**

This study longitudinally followed implants from implant placement retrospectively. The peri‐implant disease risk assessment (IDRA) tool was applied, and the performance of the Kwok and Caton (KC) implant prognostication tool was compared with that of a new tool based on the frequency of progressive bone loss (PBL). The predictive accuracy of IDRA, PBL, and KC was evaluated at the corresponding time points (T1–T5). Fisher's exact test was used to evaluate categorical variables, and the Mann‐Whitney test was used to evaluate continuous variables. Multiple regression models were built to correlate the occurrence of peri‐implantitis and implant survival. The study followed the OHstat and TRIPOD guidelines for reporting.

**Results:**

146 dental implants in 87 patients were included (age = 63.19 ± 11.38 years). IDRA classified the implants as 85.62% high‐, 14.38% moderate‐, and 0% low‐risk. The KC scores showed 7.53% of implants with a favorable‐, 65.07% with a questionable‐, and 27.4% with an unfavorable prognosis. IDRA showed limited predictive power with an AUC of 0.533 (95% CI: 0.477–0.590). The KC tool performed much better, where a score of 2 had an AUC of 0.8321 (95% CI: 0.7257–0.9386). PBL yielded a moderate but consistent effectiveness with an AUC of 0.7697 (95% CI: 0.593–0.9463).

**Conclusion:**

The KC and PBL prognostication tools exhibited good predictive capability for implant survival, while the IDRA tool demonstrated marginal efficacy in predicting the incidence of peri‐implantitis.

**Plain language summary:**

In this study, we followed 87 patients with 146 implants over time to test how well different tools can predict implant health and survival. We tested three tools, IDRA for predicting peri‐implantitis, the KC system, and a new tool based on progressive bone loss for predicting implant survival. After 2 years with the implant crowns in place, we checked for cases of peri‐implantitis. At the final follow‐up (up to 8 years), the implant survival was assessed. We found that IDRA was not effective in predicting which implants developed peri‐implantitis, even in high‐ and moderate‐risk implants. In contrast, the KC tool was much more accurate, predicting implant survival in about 80% of cases. The PBL tool was also good in predicting implant survival, but slightly less accurate than the KC tool.

## 1 INTRODUCTION

Given the irreversible nature and complex treatment of peri‐implantitis, risk assessment is considered a necessity to predict the deterioration of peri‐implant health and to evaluate the likelihood of disease progression.^1^, ^2^, ^3^ Peri‐implant mucositis, characterized by bleeding on probing (BOP), increased pocket depth (PD), and potential suppuration, often precedes crestal bone resorption that manifests as peri‐implantitis.^4^ This might significantly impact the implant survival rates.^5^

Recently, controlling risk indicators to limit the likelihood of future complications has become a key focus in implant dentistry;^6^ hence, several tools have been developed to predict peri‐implant disease risk and prognosis. The IDRA tool, developed by Heitz‐Mayfield et al., is based on peri‐implantitis as a primary endpoint. It incorporates a comprehensive evaluation of 13 variables encompassing clinical, radiographic, host‐related, and restoration quality factors to categorize the risk levels of peri‐implantitis.* Implants are categorized into three risk levels for developing peri‐implantitis: low, medium, and high.^7^ The IDRA was validated in several studies with variable success.^8^, ^9^, ^10^

Presenting as a non‐linear, accelerated, and unpredictably progressive marginal bone loss (MBL) characterized disease, peri‐implantitis might eventually lead to implant failure if not adequately dealt with.^11^ While reduced bone support often suggests compromised peri‐implant health, osseointegrated implants can maintain healthy peri‐implant tissues even with significant bone loss.^12^ With proper treatment, these implants may remain functional.^13^ Therefore, implant loss might not be a reliable indicator of prognosis, potentially leading to an overly negative assessment of lesions treated with resective or reconstructive procedures^14^ A need, therefore, arises for a structured prognostication system that elucidates the factors mentioned above and is clinically relevant, as it directly influences treatment planning.^15^

Almost two decades ago, Kwok and Caton developed a prognostication system for natural teeth using supporting tissue stability as the prognostic endpoint.^16^ Their rationale was that while tooth loss is the most definitive outcome, the follow‐up time can be lengthy, and the quality of periodontitis‐affected teeth is highly variable.^16^ Recently, Kwok and Caton developed an implant prognostication concept based on also assessing the peri‐implant supporting tissue stability. They proposed three primary categories: favorable, questionable, and unfavorable. A fourth, hopeless, was added to indicate the need to remove the implant.^17^ However, the prognostication model proposed by Kwok and Caton for implant stability was never evaluated for predictive accuracy. This is timely and crucial since it has been recently questioned whether merely the fact of a ≥ 2 mm marginal bone loss is actually a state of disease that places dental implants at increased risk of implant loss or whether this may impose an over‐diagnosis with little or no benefit.^18^ Based on observations from previous studies, it seems that the incidence of progressive bone loss may be more predictive of implant stability than the amount of bone loss evaluated at one time point.^19^

While existing periodontal prognostic systems have demonstrated promising capabilities,^20^, ^21^ a validated, comprehensive one remains elusive for implant health realms, despite ongoing efforts to develop and refine such tools. Therefore, this study aims to assess the performance of the IDRA tool, to validate the KC implant prognostication system, measure its predictive power for implant survival, and evaluate the influence of other factors on implant prognosis. Additionally, it will evaluate the predictive power of a newly developed scoring system based on progressive bone loss to assess implant survival.

## 2 MATERIALS AND METHODS

Data were collected from electronic charts of patients who received periodontal treatment between 2012 and 2024 at the University of Michigan School of Dentistry, Ann Arbor, Michigan, USA, for retrospective evaluation.All data was anonymized as per the usual IRB protocols. The study was conducted according to the Helsinki Declaration of 1975,^22^ revised in 2013.^23^ The manuscript preparation followed the 48‐point checklist provided by the OHstat guidelines.^24^, ^25^ The manuscript followed the TRIPOD checklist guideline for validating the IDRA and KC prediction models.^26^, ^27^ Ethical approval was obtained by the University of Michigan School of Medicine Institutional Review Board (IRBMED: HUM00247488).

### 2.1 Study design

The present study was designed as a longitudinal retrospective cohort study. The data collection was done at several time points as follows:

**T1**: Time of implant placement: Implant placement was done in a 2‐stage fashion without restoration or prosthetics being placed yet. Baseline demographic and health data were collected in this stage.

**T2**: Implant restoration: This is the baseline from which radiographic bone loss will be measured. This timeline was chosen to account for bone remodeling occurring in the first year due to the formation of supra‐crestal tissue attachment.^28^ The IDRA diagnostic tool was applied at this time point to evaluate the risk PI of the placed implant.

**T3**: Approximately 12 months (± 3 months) after the implant restoration (after initial remodeling).

**T4**: Around 3 years after IDRA was applied, outcomes of IDRA (occurrence of PI or lack thereof) were assessed, and the KC and PBL prognostication systems were applied.

**T5**: This occurred at the last follow‐up, when patients had a chart or radiograph taken at the University of Michigan graduate periodontics clinic. At this point, the KC and PBL assessments were performed to evaluate the ongoing success and survival or stability of the implant.

### 2.2 Inclusion and exclusion criteria

The patients were included with the following requirements:

1. Male or female patients aged ≥18 years old.
2. Patients who received at least one successful bone‐level dental implant for restoring a partially edentulous ridge
3. Patients were enrolled in a supportive periodontal therapy (SPT) program at the University of Michigan, Ann Arbor, MI.
4. The availability of patient chart records to obtain all variables listed by IDRA and KC before T1.
5. The availability of valid radiographic data (periapical or vertical bitewings) of diagnostic quality.^29^ Radiographs must be available at all times points of follow‐up (T3, T4, and T5).
6. The availability of proper periodontal clinical measurements (a baseline periodontal chart for the implant site 6–12 months post‐loading and the latest periodontal chart for the implant site as well) to provide periodontal staging and grading at the baseline and follow‐up.
7. At least 2 years of functional loading after successfully restoring the implant.

The exclusion criteria encompassed:

1. The absence of radiographs at T1, T2, T3, T4, or T5.
2. Incomplete periodontal charting at T3 or T4.
3. Missing relevant medical information or inadequate relevant implant information.
4. Radiographs that were severely angulated or of low quality.
5. Cases that had the restoration removed or changed during the follow‐up.
6. Patients not enrolled in the SPT program.

### 2.3 Data collection

Both patient and implant level data were obtained from the electronic charts of patients who fit the eligibility criteria by three examiners (M.H.A.S., E.K., H.A.). At the patient level, the following characteristics were collected: age at the time of implant placement, sex, history of periodontitis, stage and grade if diagnosed with periodontitis at the time of implant placement, DM status, smoking status (non‐smoker, former, and current smokers according to the CDC definition^30^), existence of osteoporosis, history of any bisphosphonate (intravenous or oral) use, history of radiotherapy, occlusal overload, and SPT compliance. At the implant level, the implant's brand, size, and type of implant restoration (screw or cement retained) were all collected. Patients who underwent any bone augmentation with or preceding the placement were identified. The BOP and PD (worst site) around the implants were also obtained from the periodontal chart at T0 and T1. T0 represents the baseline chart 6–12 months after implant loading, and T1 is the last available chart of the implant. Progressive PD was calculated over the follow‐up period.

### 2.4 Radiographic evaluation

The measurement of radiographic bone loss was performed on images taken at T3 with supra‐structure in place, at T4 to measure the amount of bone loss, and at T5 at the last follow‐up.^31^ The measurement performed allowed for the clear identification of the implant's platform as a reference point and distinct visualization of implant threads for the best assessment of mesial and distal bone levels in relation to the platform.^31^ A dedicated radiographic toolkit† was used to measure the bone loss around the mesial and distal sides using contrasting tools and drawing a vertical measuring line from the platform edge to the deepest visible point of marginal bone in contact with the threads. To overcome the vertical distortion, re‐calibration of the radiographic image was done depending on the actual length and diameter of the existing implant.

**FIGURE 1:**
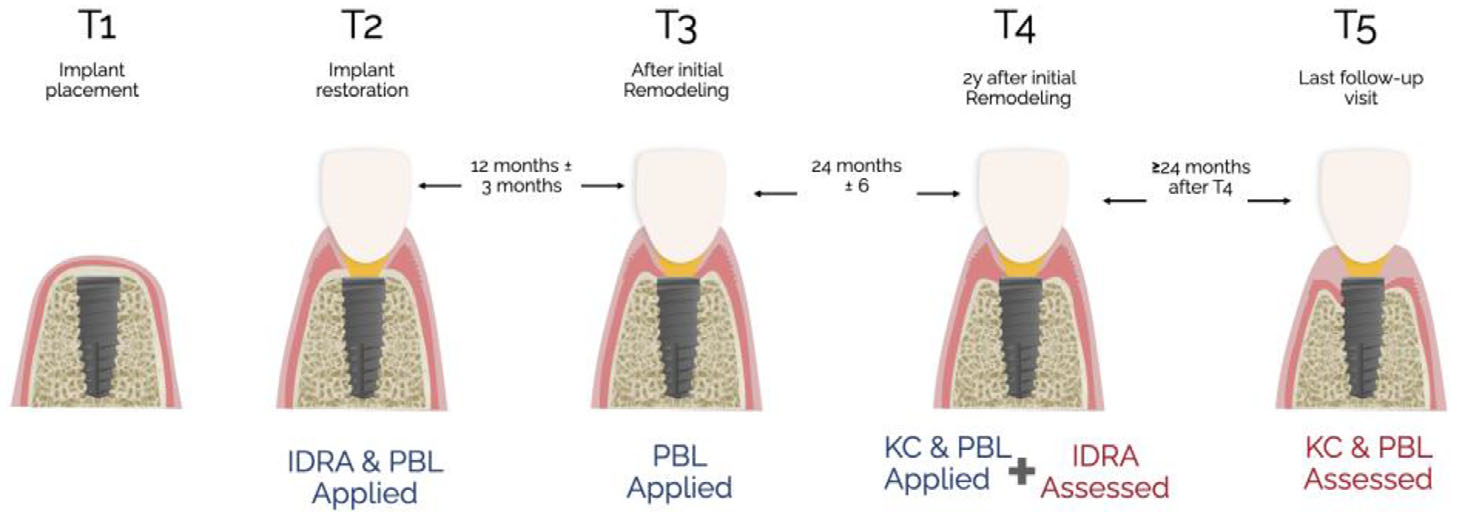
Study timeline, timing of measurement points, and application of tools.

The deepest bone resorption was chosen in the case of different resorbed bone portions. To ensure reproducibility, one examiner (E.K.) blinded to the outcome data, evaluated the radiographs. Randomly selected images (15% of the overall sample) were used to assess the examiner's intraclass correlation coefficient (ICC) by measuring all the radiographic variables twice within a period of a week. This was repeated until the examiner calibration reached an intra‐operative k‐value of 82%. The progressive marginal bone loss was then calculated over the follow‐up periods T2, T3, and T4 (Figure 1). A threshold of ≥ 1 mm bone loss was set to diagnose peri‐implantitis based on the progressive bone loss to account for possible intra‐examiner error.^32^

### 2.5 Case definitions of outcomes

#### 2.5.1 Peri‐implantitis (IDRA outcome)

Peri‐implantitis was diagnosed following the World Classification Workshop 2017 case definition.^28^ These were identified through longitudinal analysis, discriminating pathologic changes such as PD, BOP, or MBL (exceeding examiner error threshold) adjacent to the implant platform over time. This was established by contrasting the baseline clinical and radiographic parameters recorded after the prosthetic restoration of the implant (to account for bone remodeling throughout the healing phase) and data collected during the final clinical and radiographic evaluation.

#### 2.5.2 Implant failure (KC outcome)

The implant was deemed to have failed if any of these two scenarios were fulfilled:

- Implant loss is reported in the notes, chart, or radiograph.^33^
- Implant with radiographic bone loss ≥75% of the implant length at the last follow‐up.^34^

### 2.6 Assessment of prognostic tools

#### 2.6.1 Tool validation

The IDRA patient's score was calculated based on the IDRA tool's 13 baseline criteria^7^ parameters as assessed on the tool web‐based application.^*^ Namely, the history of periodontitis, percentage of sites with BOP, prevalence of PD ≥ 5 mm, bone loss related to the patient's age, periodontal staging and grading, and SPT compliance, distance between the margin of the prosthesis and the marginal bone level assessed on the radiograph after implant loading, and prosthetic factors such as implant–prosthesis precision of adaptation and the presence of excess cement.^35^ The scores obtained were attributed to a value of low, medium, or high. The cohort cases were assessed for the incidence of PI after at least 2 years post‐loading the implant, according to the World Classification Workshop 2017.^12^

#### 2.6.2 Prognostication system predictive power

The KC score was calculated based on 10 variables (one local (plaque control), five general (presence of periodontitis, adherence to supportive care, smoking, diabetes mellitus, osteoporosis), four other factors (bisphosphonates, radiation therapy, occlusion, genetic factors). Score levels from 1 to 4 were assigned as follows: 1 = favorable, 2 = questionable, 3 = unfavorable, 4 = hopeless.

Since the authors of the KC tool did not assign weights/values to their suggested parameters, we assigned a simple scoring system of scores 1–3 (1 being lowest, 3 being highest) based on the severity of the variable. Based on the total score for each implant, the following prognoses were assigned (Table S1 in the online *Journal of Periodontology*):

**Low risk**: favorable prognosis (scores of 10–15)

**Medium risk**: questionable prognosis (scores of 16–23)

**High risk**: unfavorable prognosis (scores of 24–30)

**Table** **S1** demonstrates all variables included in, weights and scores assigned to KC tool.

#### 2.6.3 PBL for implant survival

Progressive bone loss was defined as additional radiographic bone loss (of ≥1 mm) between each of the three visits, similar to:

**T2 compared with T1**: marginal bone loss at implant restoration compared with implant placement

**T3 compared with T2**: marginal bone loss approximately 12 months after the implant restoration

**T4 compared with T3**: Around 3 years after the implant restoration.

Reminiscent of the landmark study by Lang and co‐workers regarding BOP as a predictor for the progression of periodontitis,^36^ the values were assigned as follows: 1 for 1/3 visits, 2 for 2/3, and 3 for 3/3 with ≥1 mm compared with the previous visit.

### 2.7 Statistical analysis

All analyses were performed using specialized statistics software.‡ Demographic and clinical variables were analyzed and synthesized as percentages for categorical variables and as means with standard deviations for continuous variables, a value of *p* < 0.05 was set as the threshold for statistical significance. Comparisons between the occurrences of outcomes in the different categorical variables were performed using Fisher's exact test, while the Mann–Whitney test was used for continuous variables.

Multilevel logistic regression models were built to analyze the association between the IDRA score categories and the occurrence of peri‐implantitis at the 2‐year follow‐up. The magnitude of such an association is quantified by odds ratios (OR) to indicate the system's reliability. The association between the KC prognostication score level (1–4) and both implant survival and bone loss (measured in %) were statistically analyzed to assess the discriminatory ability of the KC prognostication score to predict implant survival and failure compared with the bone loss prediction ability for implant survival and failure.

Predicted probabilities were obtained for the unit of analysis (implant‐level). The predictive performance of these models was assessed by calculating the area under the receiver operating characteristic (ROC) curve (AUC‐ROC). The associations between the score levels and both implant survival and bone loss (measured in mm) were reported by odd ratios, while the comparison between the predictive powers was reported by measuring the AUC‐ROC.

To determine the necessary sample size for the external validation of the IDRA tool in predicting peri‐implantitis, a sample size estimation was performed using a multilevel logistic regression model. Based on an estimated event rate of 15%, an OR of 2.0, and an ICC of 0.10, a standard logistic regression approach indicated that a minimum of 128 implants would be required. After accounting for clustering effects within patients, the required sample size increased to 142 implants.

## 3 RESULTS

### 3.1 Characteristics of the study population

A total number of 146 implants in 87 patients, 37 males (42.52%) and 50 females (57.47%) with a mean age of 63.19 ± 11.38 years were included in the present investigation. The mean follow‐up was 7.86 ± 2.74 years. Over half of the patients were non‐smokers (54%). Comorbid conditions were present in a minority of subjects, with diabetes in 16.1% and bisphosphonate use in 9.2%, and most patients (83.9%) had undergone some sort of treatment for periodontitis. According to the 2017 World Workshop classification,^12^ most patients were categorized in the early stages of the disease, with 14.9% in stage 1 and 39.1% in s tage 2. A demographic table of characteristics of patients included in the study is discussed in Table 1. Table S2 in the online *Journal of Periodontology* demonstrates the initial scoring and prognosis assignment to IDRA, KC, and PBL.

**TABLE 1:** Demographic table of characteristics of patients included in the study.

Variables	Categories	*N*	Percentage
**Patient‐level**			
No. of patients		87	
Mean age		63.19 ± 11.38	
Follow‐up time		7.86 ± 2.74	
Sex	Male		42.52
	Female		57.47
No. of implants		146	
Site	Anterior		3.44
	Posterior		96.55
Heavy smokers	Current	5	5.74
	Former	26	29.88
Light smokers	Current	3	3.44
	Former	6	6.89
Non‐smokers	Yes	47	54.02
Diabetes	Yes	14	16.09
	No	73	83.90
Bisphosphonate therapy	Yes	8 (1 Iv; 7 oral)	9.19
Radiation therapy	No	83	95.4
Osteoporosis	No	80	91.95
History of periodontitis treatment	Yes	73	83.90
	No	14	16.09
Adherence to SPT	<5 months	17	11.64
	6 months	26	17.80
	Casual	40	27.39
Occlusal overload	Yes	44	50.57
	No	41	47.12

2017 World Workshop Classification of Patients, *N* (%)	Stage	Grade A	Grade B	Grade C
	1	13 (14.94%)	0	0
	2	34 (39.08%)	24 (27.58%)	0
	3	0	12 (13.79%)	1 (1.14%)
	4	0	0	3 (3.44%)

Variables	Categories	*N*	Percentage
**Implant‐level**			
PD	>5 mm	656 sites	
BOP	Yes	1959	
Implant location	Maxilla	58	39.7
	Mandible	88	60.27
Restorative margin	<1.5 mm	68	46.57
	>1.5 mm	78	53.42
Retention	Cement‐retained	109	74.65
	Screw‐retained	37	25.34
Prosthetic component	Cleanable	59	52.2
	Uncleanable	30	26.5
Subgingival margins	Yes	51	34.93
	No	50	34.24

### 3.2 Marginal bone loss

Table 2 illustrates the marginal bone loss (MBL) over the study period. Over a 2‐year period, MBL was categorized as > 2 mm or < 2 mm. Specifically, 41 cases (28.08%) exhibited MBL values greater than 2 mm, with measurements ranging from 2.005 to 4.53 mm, while 105 cases (71.91%) exhibited MBL values less than 2 mm, ranging from 0 to 1.94 mm. At the last follow‐up visit, 49 cases (33.56%) demonstrated MBL exceeding 2 mm, with values ranging from 2.005 to 8.215 mm, and 97 cases (66.43%) demonstrated MBL less than 2 mm, with a range of 0 to 1.96 mm. Supplementary Table 3 in the online *Journal of Periodontology* shows the magnitude of MBL around implants in 10% increments at the end of the follow‐up.

**TABLE 2:** MBL outcomes.

Variables	Categories	*N*	%
Marginal bone loss during a 2‐year period	>2 mm	41 (2.005–4.53 mm)	28.08
	<2 mm	105 (0–1.94 mm)	71.91
Marginal bone loss at the last follow‐up visit	>2 mm	49 (2.005–8.215 mm)	33.56
	<2 mm	97 (0–1.96 mm)	66.43

### 3.3 IDRA score and peri‐implantitis

**FIGURE 2:**
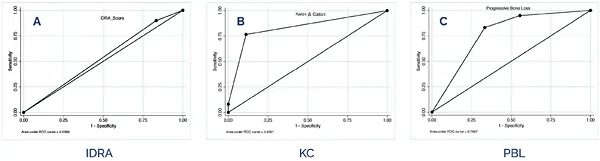
The predictive capability of IDRA, KC, and PBL using the area under the ROC curve (AUC).

Two years after implant placement, 45 out of the 125 implants with a high IDRA score developed peri‐implantitis (36%), while the remaining 80 did not (64%). In contrast, among the 21 implants with a moderate risk, only five developed PI (23.8%) (Table 3). The association between the IDRA score and PI risk was analyzed using OR. The analysis reported an OR of 1.8 (*p* = 0.281) The area under the ROC curve (AUC) was analyzed, yielding a value of 0.533 (± 0.0287 95%, CI 0.47705‐0.58962) (Figure 2A). No statistically significant difference between the groups with high or moderate IDRA scores was found for the risk of developing peri‐implantitis.

**TABLE 3:** Cross table of IDRA score with PI and of K&C score with implant failure.

	Yes	No	Total
**Peri‐implantitis — Risk assessment**			
IDRA score			
High	45 36%	80 64%	125 100%
Moderate	5 23.80%	16 76.19%	21 100%
Total	50 34.24%	96 65.75%	146 100%
**Implant failure — prognostication**			
K&C score			
1	11 100%	0	11 100%
2	94 98.9%	1 1,05%	95 100%
3	32 80%	8 20%	40 100%
Total	137 93.83%	9 6.16%	146 100%

### 3.4 KC score and implant survival

All implants that were assigned a score of 1, survived (100%). Among the 95 implants with a score of 2, only one implant did not survive (1.05%). In contrast, for the implants with a score of 3, eight implants did not survive (20%) (Table 3). The analysis reported an OR of 1 for a KC score of 1 and 3. However, for a KC score of 2, the OR was 23.5 (*p* = 0.003). The AUC was analyzed, yielding a value of 0.8321 (± 0.0543 95%, C.I 0.72568‐0.93855) (Figure 2B). This indicates that the KC prognostic tool has a good discriminatory ability to distinguish between implant survival and failure based on the assigned scores.

Progressive bone loss was seen at 29 sites (19.86%) (1.055‐4.975 mm) and 117 sites (80.13%) (−1.795‐0.97 mm) with a loss of less than 1 mm. To assess whether bone loss has discriminatory power for implant survival, the AUC was calculated, resulting in a value of 0.7697 (± 0.0901 95% CI 0.59303‐0.94631) (Figure 2C). The AUC for the KC tool is higher, suggesting that the KC tool has greater prognostic power than bone loss in predicting implant survival (Supplemental Table 4). In addition to the asymptotic confidence interval initially reported (AUC = 0.770; 95% CI: 0.593–0.946), we performed both non‐parametric and cluster bootstrap analyses with 1000 resamples. The standard bootstrap confirmed the robustness of the results (AUC = 0.770; 95% CI: 0.581–0.951). To account for the correlation of multiple implants within the same patient, we further applied a cluster bootstrap by patient, which yielded a similar discriminative ability (AUC = 0.770) with a 95% cluster bootstrap confidence interval of 0.555–0.978.

## 4 DISCUSSION

This comprehensive study assessed the IDRA scores as a risk assessment tool for peri‐implantitis, the KC prognostication tool for implant survival over a follow‐up of 8 years. After 2 years, in the presence implant crown restoration, the incidence of peri‐implantitis was evaluated. At the last follow‐up, implant survival was assessed. Our data showed no statistically significant relationship between the IDRA score and peri‐implantitis for both high and moderate‐risk implants with an OR of 1.8. The second aspect examined in our study was the correlation between the KC prognostication tool after 3 years of loading and the prediction of implant survival. To further compare the amount of bone loss after 2 years of loading, we used a progressive bone loss measure at different thresholds as an indicator of implant stability. We found that the KC systems showed a predictability rate of 80%.

These findings are consistent with De Ry et al.’s study,^8^ which focused on the IDRA tool's ability to predict peri‐implantitis in patients with periodontitis treated with implant‐supported fixed dental prostheses after 5 years or more of loading, revealing that peri‐implantitis was present in 12% of patients at moderate risk and in 27% at high IDRA risk. The comparison of the OR for PI development between patients with high IDRA risk compared with patients with moderate IDRA risk was 2.727, with no statistically significant difference between the groups.^8^

Recently, Rebeiz et al. demonstrated a significant association between IDRA risk levels and the incidence of peri‐implantitis (*p* = 0.003).^36^ The study indicated that high‐risk patients had 5.2 times higher odds of developing peri‐implantitis than low‐to‐moderate‐risk patients (*p* < 0.001). Moreover, the study emphasized that the potential use of this tool can be considered before implant placement and during the periodontal maintenance phase, thus highlighting its practical fit.^36^

Among all the implants analyzed in our study, no implant was classified as low‐risk. Similarly, De Ry et al.^8^ had excluded all the low‐risk implant patients in their study. On the other hand, Rebeiz et al.^37^ included low‐risk implants as one of the categories that the high‐risk implants were compared with, demonstrating a significant difference of *p*‐value < 0.001.

Analysis of the KC tool revealed an AUC of 0.8321 indicating good discriminatory power in predicting implant survival. This result was compared with progressive bone loss, which yielded an AUC of 0.7697. This comparison suggested that while PBL predicts implant survival well, the KC tool has superior performance. The notion that PBL (at each follow‐up point) may act as a good predictor for peri‐implantitis as well as implant survival was based on observations from previous studies.^38^, ^39^ Though easy to use, this method involves a “wait and see” approach rather than a real prognostic tool used at the baseline (patient presentation) like the KC tool.

Peri‐implant inflammations have a wide prevalence range, ultimately leading to implant loss without multifaceted prevention and therapy concepts.^40^, ^41^ While several systemic, local, and individual factors may contribute to peri‐implantitis, there is no consistency in the reported prevalence in the literature, owing to a lack of structured classification systems. Local predisposing factors and systemic drivers increases the risk of peri‐implantitis and implant loss^42^ and emerging evidence is progressively revealing further systemic and local associations.^43^, ^44^ This calls for updating current valuable tools such as IDRA to enhance the risk assessment process.

Decker et al. in 2015^45^, conducted a database search to compile articles to test for the proposed prognostic scale, which constituted the existing classification by Froum and Rosen^46^, and the therapeutic decision tree by Okayasu and Wang.^47^ The prognostic categories were also related to implant stability derived from the KC prognostication for teeth. The authors suggested that an initial provisional prognosis may be accurate, and re‐assignment of a prognosis should be done after treatment.^45^

Another system reviewed evidence‐based factors based on a blind survey of specialists from three dental schools. In addition to the factors discussed, they also included “excess cement” as one of the important considerations. The authors mentioned, however, that this was the first attempt to propose a comprehensive prognostication system, and further validation studies are needed.^48^

Although the study is one of the first to assess the KC system for implant prognosis and to compare it with a simple exercise of progressive bone loss, its limitations are worth acknowledging. First, its retrospective design may raise potential biases associated with the data collection methodology. We had multiple clinicians with different skill levels, and the procedures were not standardized. Also, the study was conducted at a single institute, which limits the generalization of results to other populations. The reliance on electronic health records, documented by different providers, further compromises their generalizability, as missing information can impact accurate case classification. Additionally, the patient cohort includes individuals with varying medical histories who received the implant placement using different implant systems, with or without simultaneous grafting. Given these factors and the absence of external validation, the results should be interpreted with caution, and future multi‐center investigations will be imperative to confirm their generalizability.

To overcome the impact of these limitations and to enhance the data variability, we established a threshold of errors to exclude any measurements below 1 mm, which ensured better bone level assessment.^49^ Furthermore, the evaluation criteria across the medical records were standardized during the data collection. Additionally, we used the same group of patients for assessing IDRA and the KC systems, ensuring a different baseline date for scoring of the two, by a single examiner, which makes the results of the two comparable. Our sample size of 146 implants, compared with the other studies with a smaller number of implants included,^8^ provides a more robust database for assessing the relationship between risk factors and peri‐implantitis.

Future studies should prioritize multi‐center validation with necessary modifications of the tools, to ensure feasibility in routine practice. KC can be used at the first visit after delivery of the definitive restoration to stratify the implant survival risk. Higher scores will prompt closer surveillance, more emphasis on risk‐factor control, potential prosthetic adjustments, etc. KC is a single‐time assessment that is fast and compatible with standard clinical assessments.

In contrast, progressive bone loss can work as a standalone tool or complement KC by monitoring disease activity over time. Since PBL reflects a trajectory of bone loss rather than a static defect, it distinguishes between stable and progressive conditions, thereby providing valuable guidance on the timing and necessity of surgical intervention.

## 5 CONCLUSION

Within the limitations of this study, the KC prognostication tool exhibited superior predictive capability for implant survival, in comparison with bone loss as a prognostic tool. In contrast, the IDRA tool demonstrated marginal efficacy in anticipating the onset of peri‐implantitis.

## AUTHOR CONTRIBUTIONS

**Muhammad H. A. Saleh**: Conceptualization (lead); methodology (lead); data curation (support); manuscript draft review (Lead). **Era Kakar**: Investigation (equal); writing the manuscript draft (equal). **Giuseppe Troiano**: Formal analysis (lead). **Hamzeh Almashni**: Investigation (equal); writing the manuscript draft (equal). **Jonathan Misch**: Data curation (lead). **Zoltán Baráth**: Project management (support); manuscript draft review (support). **Fariba Esperouz**: Formal analysis (supporting); writing the manuscript draft (support). **Shahad Alhazmi**: Writing the manuscript draft (support). **Hom‐Lay Wang**: Manuscript draft review (support). **Istvan Urban**: Conceptualization (support); manuscript draft review (support). All authors approved the manuscript and are considered accountable for the study content.

## CONFLICT OF INTEREST STATEMENT

The authors declare no conflicts of interest.

## Supporting information



Supporting Information

## Data Availability

The data that support the findings of this study are available upon request from the corresponding author. The data are not publicly available due to privacy or ethical restrictions.

## Footnotes

* Perio‐Tools. Implant Disease Risk Assessment (IDRA). Perio‐Tools website: https://www.perio‐tools.com/idra/en/. Accessed August 24, 2025.

† Dental Enterprise Viewer, Medicore Imaging, Charlotte, NC, USA.

‡ Stata software, Stata Corporation, College Station, TX, USA.

## References

[1] Fu JH , Wang HL . Breaking the wave of peri‐implantitis. Periodontol 2000. 2020;84:145‐160. 32844418 10.1111/prd.12335

[2] Heitz‐Mayfield LJA . Disease progression: identification of high‐risk groups and individuals for periodontitis. J Clin Periodontol. 2005;32:196‐209. 16128838 10.1111/j.1600-051X.2005.00803.x

[3] Lang NP , Suvan JE , Tonetti MS . Risk factor assessment tools for the prevention of periodontitis progression a systematic review. J Clin Periodontol. 2015;42:S59‐S70. 25496279 10.1111/jcpe.12350

[4] Lindhe J , Meyle J . Periodontology peri‐implant diseases: Consensus Report of the Sixth European Workshop on Periodontology. J Clin Periodontol. 2008;35:282‐285. 18724855 10.1111/j.1600-051X.2008.01283.x

[5] Lang NP , Bosshardt DD , Lulic M . Do mucositis lesions around implants differ from gingivitis lesions around teeth? J Clin Periodontol. 2011;38:182‐187. 21323714 10.1111/j.1600-051X.2010.01667.x

[6] Heitz‐Mayfield LJ , Needleman I , Salvi GE , Pjetursson BE . Consensus statements and clinical recommendations for prevention and management of biologic and technical implant complications. Int J Oral Maxillofac Implants. 2014;29:346‐350. 24660208 10.11607/jomi.2013.g5

[7] Heitz‐Mayfield LJA , Heitz F , Lang NP . Implant disease risk assessment IDRA–a tool for preventing peri‐implant disease. Clin Oral Implants Res. 2020;31:397‐403. 32003037 10.1111/clr.13585

[8] De Ry SP , Roccuzzo A , Lang NP , et al. Evaluation of the Implant Disease Risk Assessment (IDRA) tool: a retrospective study in patients with treated periodontitis and implant‐supported Fixed Dental Prostheses (FDPs). Clin Oral Implants Res. 2021;32:1299‐1307. 34388276 10.1111/clr.13828 PMC9290928

[9] Bornes R , Montero J , Ferreira A , Rosa N , Correia A . Dentists' perceptions and usability testing of the implant disease risk assessment IDRA, a tool for preventing peri‑implant disease: a qualitative study. J Dent. 2023;136:104630. 37488043 10.1016/j.jdent.2023.104630

[10] de Medeiros Dantas JL , Freire GCB , Dos Santos Calderon P , Duarte PM , de Vasconcelos Gurgel BC . Retrospective assessment of patients' risk for peri‐implant diseases using the Implant Disease Risk Assessment (IDRA) tool: a cohort study. Clin Implant Dent Relat Res. 2024;26(5):1056‐1066. doi:10.1111/cid.13371 39113398

[11] Derks J , Schaller D , Håkansson J , Wennström JL , Tomasi C , Berglundh T . Peri‐implantitis – onset and pattern of progression. J Clin Periodontol. 2016;43:383‐388. 26900869 10.1111/jcpe.12535

[12] Berglundh T , Armitage G , Araujo MG , et al. Peri‐implant diseases and conditions: consensus report of workgroup 4 of the 2017 World Workshop on the Classification of Periodontal and peri‐Implant Diseases and Conditions. J Periodontol. 2018;89:S313‐S318. 29926955 10.1002/JPER.17-0739

[13] Ravida A , Siqueira R , Saleh I , Saleh MHA , Giannobile A , Wang HL . Lack of clinical benefit of implantoplasty to improve implant survival rate. J Dent Res. 2020;99:1348‐1355. 32718212 10.1177/0022034520944158

[14] Monje A , Nart J . Management and sequelae of dental implant removal. Periodontol 2000. 2022;88:182‐200. 35103326 10.1111/prd.12418

[15] Saleh MHA , Dias DR , Kumar P . The economic and societal impact of periodontal and peri‐implant diseases. Periodontol 2000. 2024. doi:10.1111/prd.12568 PMC12842886 38693603

[16] Kwok V , Caton JG . Commentary: prognosis revisited: a system for assigning periodontal prognosis. J Periodontol. 2007;78:2063‐2071. 17970671 10.1902/jop.2007.070210

[17] Kwok V , Caton JG , Hart ID , Kim T‐S . Dental implant prognostication: a commentary. J Periodontol. 2023;94:713‐721. 36740787 10.1002/JPER.22-0196

[18] Albrektsson T , Tengvall P , Amengual‐Penafiel L , Coli P , Kotsakis G , Cochran DL . Implications of considering peri‐implant bone loss a disease, a narrative review. Clin Implant Dent Relat Res. 2022;24:532‐543. 35639515 10.1111/cid.13102 PMC9542069

[19] Ravida A , Galli M , Siqueira R , Saleh MHA , Galindo‐Moreno P , Wang HL . Diagnosis of peri‐implant status after peri‐implantitis surgical treatment: proposal of a new classification. J Periodontol. 2020;91:1553‐1561. 32449808 10.1002/JPER.20-0124

[20] Saleh MHA , Dukka H , Troiano G , et al. External validation and comparison of the predictive performance of 10 different tooth‐level prognostic systems. J Clin Periodontol. 2021;48:1421‐1429. 34472120 10.1111/jcpe.13542

[21] Saleh MHA , Dukka H , Troiano G , et al. Long term comparison of the prognostic performance of PerioRisk, periodontal risk assessment, periodontal risk calculator, and staging and grading systems. J Periodontol. 2022;93:57‐68. 33914347 10.1002/JPER.20-0662

[22] World Medical Association . World Medical Association Declaration of Helsinki: Ethical principles for medical research involving human subjects. Accessed March 23, 2023. Available at: https://www.wma.net/policies-post/wma-declaration-of-helsinki-ethical-principles-for-medical-research-involving-human-subjects/

[23] World Medical A . World Medical Association Declaration of Helsinki: ethical principles for medical research involving human subjects. JAMA 2013;310:2191‐2194. 24141714 10.1001/jama.2013.281053

[24] Best AM , Lang TA , Greenberg BL , et al. The OHStat Guidelines for Reporting Observational Studies and Clinical Trials in Oral Health Research: explanation and elaboration. J Am Dent Assoc. 2024;155:e1‐e21. 39001723 10.1016/j.adaj.2024.06.007

[25] Best AM , Lang TA , Greenberg BL , et al. The OHStat Guidelines for Reporting Observational Studies and Clinical Trials in Oral Health Research: manuscript checklist. J Am Dent Assoc. 2024;155:708‐714. 39001724 10.1016/j.adaj.2024.06.006

[26] Collins GS , Reitsma JB , Altman DG , Moons KG . Transparent Reporting of a multivariable prediction model for Individual Prognosis or Diagnosis (TRIPOD): the TRIPOD statement. Ann Intern Med. 2015;162:55‐63. 25560714 10.7326/M14-0697

[27] Moons KG , Altman DG , Reitsma JB , et al. Transparent reporting of a multivariable prediction model for Individual Prognosis or Diagnosis (TRIPOD): explanation and elaboration. Ann Intern Med. 2015;162:W1‐73. 25560730 10.7326/M14-0698

[28] Renvert S , Persson GR , Pirih FQ , Camargo PM . Peri‐implant health, peri‐implant mucositis, and peri‐implantitis: case definitions and diagnostic considerations. J Periodontol. 2018;89(1):S304‐S312. 29926953 10.1002/JPER.17-0588

[29] Prichard JF . Interpretation of radiographs in periodontics. Int J Periodontics Restorative Dent. 1983;3:8‐39. 6574120

[30] National Center for Health Statistics . Table A–12a. Age‐adjusted percentages (with standard errors) of current cigarette smoking status among adults aged 18 and over, by selected characteristics: United States, 2018. Summary Health Statistics Tables: National Health Interview Survey, 2018. Accessed October 26, 2025. https://ftp.cdc.gov/pub/Health_Statistics/NCHS/NHIS/SHS/2018_SHS_Table_A-12.pdf

[31] Sahrmann P , Kuhl S , Dagassan‐Berndt D , Bornstein MM , Zitzmann NU . Radiographic assessment of the peri‐implant site. Periodontol 2000. 2024;95:70‐86. 38951952 10.1111/prd.12577

[32] García‐García M , Mir‐Mari J , Benic GI , Figueiredo R , Valmaseda‐Castellõn E . Accuracy of periapical radiography in assessing bone level in implants affected by peri‐implantitis: a cross‐sectional study. J Clin Periodontol. 2016;43:85‐91. 26660842 10.1111/jcpe.12491

[33] Simonis P , Dufour T , Tenenbaum H . Long‐term implant survival and success: a 10‐16‐year follow‐up of non‐submerged dental implants. Clin Oral Implants Res. 2010;21:772‐777. 20636731 10.1111/j.1600-0501.2010.01912.x

[34] Greenstein G , Cavallaro J . Failed dental implants: diagnosis, removal and survival of reimplantations. J Am Dent Assoc. 2014;145:835‐842. 25082932 10.14219/jada.2014.28

[35] De Ry SP , Roccuzzo A , Lang NP , et al. Evaluation of the implant disease risk assessment (IDRA) tool: a retrospective study in patients with treated periodontitis and implant‐supported fixed dental prostheses (FDPs). Clinical Oral Implants Research. 2021;32:1299‐1307. 34388276 10.1111/clr.13828 PMC9290928

[36] Lang NP , Joss A , Orsanic T , Gusberti FA , Siegrist BE . Bleeding on probing. A predictor for the progression of periodontal disease? J Clin Periodontol. 1986;13:590‐596. 3489010 10.1111/j.1600-051x.1986.tb00852.x

[37] Rebeiz T , Nasr L , Kassir AR , Menassa G , Chakar C . Assessment of the association between the Implant Disease Risk Assessment (IDRA) tool and peri‐implantitis: a retrospective cohort study with up to 8 years of follow‐up. Int J Oral and Maxillofac Surg. 2024;53:845‐852. 38806315 10.1016/j.ijom.2024.05.002

[38] Rodriguez MV , Ravida A , Saleh MHA , et al. Is the degree of physiological bone remodeling a predictive factor for peri‐implantitis? J Periodontol. 2022;93:1273‐1282. 35536150 10.1002/JPER.21-0723 PMC9796402

[39] Ravida A , Samal A , Qazi M , et al. Interproximal implant thread exposure after initial bone remodeling as a risk indicator for peri‐implantitis. J Periodontol. 2023;94:751‐764. 36576085 10.1002/JPER.22-0499

[40] Smeets R , Henningsen A , Jung O , Heiland M , Hammächer C , Stein JM . Definition, etiology, prevention and treatment of peri‐implantitis–a review. Head Face Med. 2014;10:34. 25185675 10.1186/1746-160X-10-34 PMC4164121

[41] Derks J , Tomasi C . Peri‐implant health and disease. A systematic review of current epidemiology. J Clin Periodontol. 2015;42(16):S158‐171. 25495683 10.1111/jcpe.12334

[42] Monje A , Kan JY , Borgnakke W . Impact of local predisposing/precipitating factors and systemic drivers on peri‐implant diseases. Clin Implant Dent Relat Res. 2023;25:640‐660. 36533411 10.1111/cid.13155

[43] Almashni H , Kakar E , Nava P , Wang HL , Saleh MHA . Influence of rheumatoid arthritis on peri‐implant diseases: a longitudinal retrospective clinical and radiographic evaluation. J Periodontol. 2025;96(8):933‐943. doi:10.1002/JPER.24-0376 39812457 PMC12424578

[44] Misch J , Alrmali AE , Galindo‐Fernandez P , Saleh MHA , Wang HL . Peri‐implant tissue stability: the PROS concept. Int J Oral Implantol (Berl). 2025;18:73‐84. 40047364

[45] Decker AM , Sheridan R , Lin GH , Sutthiboonyapan P , Carroll W , Wang HL . A prognosis system for periimplant diseases. Implant Dent. 2015;24:416‐421. 26035268 10.1097/ID.0000000000000276

[46] Rosen PS , Froum SJ , Sarmiento H , Wadhawani CP . A revised peri‐implantitis classification scheme: adding three‐dimensional considerations to facilitate prognosis and treatment planning. Int J Periodontics Restorative Dent. 2022;42:291‐299. 35472104 10.11607/prd.5876

[47] Okayasu K , Wang HL . Decision tree for the management of periimplant diseases. Implant Dent. 2012;21(3):253. doi:10.1097/ID.0b013e3182263589 21778886

[48] Najafi B , Kheirieh P , Torabi A , Cappetta EG , Najafi A , Singh SM . A new prognostication system for dental implants. Int J Periodontics Restorative Dent. 2018;38:e17‐e24. 29240215 10.11607/prd.2996

[49] Walton TR , Layton DM . Intra‐ and inter‐examiner agreement when assessing radiographic implant bone levels: differences related to brightness, accuracy, participant demographics and implant characteristics. Clin Oral Implants Res. 2018;29:756‐771. 30240063 10.1111/clr.13290

