# Supplementary material for: Predicting peri‐implantitis incidence and implant failure via risk‐assessment and prognostication tools: A validation study
Source: J Periodontol. 2025 Dec 26;97(8):1784–94. doi: 10.1002/jper.70047 (PMC13502412; doi:10.1002/jper.70047)
Supplement: Supplementary file 1 [file JPER-97-1784-s001.docx]

**Legend:**

**Supplemental Table 1:** Weights and scores assigned to KC tool

**Supplemental Table 2:** Initial scoring and prognosis assignment

**Supplemental Table 3:** Alveolar Bone loss around implants

**Supplemental Table 4:** KC Predictions showing higher specificity, while PBL has higher sensitivity.

| **Variable** | **Score** | **1** | **2** | **3** |
| --- | --- | --- | --- | --- |
| **Plaque Control** | | Plaque score <20% | Plaque score ≥20-50% | Plaque score ≥50% |
| **Presence of Periodontitis** | | No periodontitis | Stage I-II periodontitis | Stage III-IV periodontitis |
| **Adherence to Supportive Care** | | Non-Complier | Erratic complier | Complier |
| **Smoking** | | Non-smoker | Former smoker | Current light |
| **Diabetes Mellitus** | | No diabetes | Well-controlled diabetes | Poorly controlled diabetes |
| **Osteoporosis** | | No osteoporosis | Osteopenia (pre-osteoporosis), no dental impact | Osteoporosis, some dental impact |
| **Bisphosphonates** | | Never used | Short-term use Oral only | Long-term oral use OR IV |
| **Radiation Therapy** | | No history of radiation therapy | Past radiation, minimal to no impact on oral health | Radiation with moderate impact on oral health |
| **Occlusion** | | Normal occlusion | Slight occlusal issues | Significant occlusal issues affecting teeth previously |
| **Genetic Factors** | | No known genetic predisposition affecting dental health | Mild genetic risk factors | Moderate genetic risk factors with previous impact on dental health |
| **Supplemental Table 1: Weights and scores assigned to KC tool** | | | | |

| **IDRA** | High | 85.62% |
| --- | --- | --- |
|  | Moderate | 14.38% |
| **KC** | 1 | 7.53% |
|  | 2 | 65.07% |
|  | 3 | 27.4% |
| **PBL** | 1 | 80.13% |
|  | 2 | 12.32% |
|  | 3 | 7.53% |
| **Supplemental Table 2: Initial scoring and prognosis assignment** | | |

| **No. of patients** | **% MBL around implant** | **N** | **%** |
| --- | --- | --- | --- |
| **Alveolar Bone Loss (%)** | 10 | 13 | 14.94 |
|  | 20 | 17 | 19.54 |
|  | 30 | 28 | 32.18 |
|  | 40 | 13 | 14.94 |
|  | 50 | 9 | 10.34 |
|  | 60 | 3 | 3.44 |
|  | 70 | 1 | 1.14 |
|  | 80 | 1 | 1.14 |
|  | 100 | 2 | 2.29 |
| **Supplemental Table 3: Alveolar Bone loss around implants.** MBL: Marginal bone loss | | | |

| **Model** | **AUC** | **Cut-off** | **Sensitivity** | **Specificity** | **PPV** | **NPV** |
| --- | --- | --- | --- | --- | --- | --- |
| **PBL** Predictions | 0.770 | 0.974 | 0.832 | 0.667 | 0.974 | 0.207 |
| **KC** Predictions | 0.832 | 0.990 | 0.766 | 0.889 | 0.991 | 0.200 |

**Supplemental Table 4:** KC Predictions showing higher specificity, while PBL has higher sensitivity.
